# Radiofrequency Electromagnetic Radiation and Memory Performance: Sources of Uncertainty in Epidemiological Cohort Studies

**DOI:** 10.3390/ijerph15040592

**Published:** 2018-03-26

**Authors:** Christopher Brzozek, Kurt K. Benke, Berihun M. Zeleke, Michael J. Abramson, Geza Benke

**Affiliations:** 1 Centre for Population Health Research on Electromagnetic Energy (PRESEE), School of Public Health and Preventive Medicine, Monash University, 553 St Kilda Road, Melbourne, VIC 3004, Australia; berihun.zeleke@monash.edu (B.M.Z.); michael.abramson@monash.edu (M.J.A.); geza.benke@monash.edu (G.B.); 2 School of Engineering, University of Melbourne, Parkville, Melbourne, VIC 3010, Australia; kbenke@unimelb.edu.au or kurt.benke@ecodev.vic.gov.au; 3 Department of Economic Development, Jobs, Transport and Resources (DEDJTR), AgriBio Centre, 5 Ring Rd, Bundoora, Melbourne, VIC 3083, Australia

**Keywords:** radiofrequency electromagnetic radiation, epistemic uncertainty, aleatory uncertainty, memory, cognitive function

## Abstract

Uncertainty in experimental studies of exposure to radiation from mobile phones has in the past only been framed within the context of statistical variability. It is now becoming more apparent to researchers that epistemic or reducible uncertainties can also affect the total error in results. These uncertainties are derived from a wide range of sources including human error, such as data transcription, model structure, measurement and linguistic errors in communication. The issue of epistemic uncertainty is reviewed and interpreted in the context of the MoRPhEUS, ExPOSURE and HERMES cohort studies which investigate the effect of radiofrequency electromagnetic radiation from mobile phones on memory performance. Research into this field has found inconsistent results due to limitations from a range of epistemic sources. Potential analytic approaches are suggested based on quantification of epistemic error using Monte Carlo simulation. It is recommended that future studies investigating the relationship between radiofrequency electromagnetic radiation and memory performance pay more attention to treatment of epistemic uncertainties as well as further research into improving exposure assessment. Use of directed acyclic graphs is also encouraged to display the assumed covariate relationship.

## 1. Introduction

Mobile (cellular) phone use and subsequent exposure to radiofrequency electromagnetic radiation (RF-EMR) via associated wireless technologies has become ubiquitous in the Western world with an estimated 6.9 billion subscriptions globally in 2014 [1]. The steady proliferation of RF-EMR- emitting devices has raised concerns about potential adverse health effects from members of the general public, as well as expert groups such as the Scientific Committee on Emerging and Newly Identified Health Risks (SCENIHR) and the World Health Organization (WHO) [2,3]. Many studies have been conducted investigating the potential health effects of RF-EMR exposure; still the results have largely been inconsistent. Based on the results of the Interphone study [4], the International Agency for Research on Cancer (IARC) classified RF-EMR as a group 2B possible carcinogen [5]. One growing area of concern is the impact RF-EMR exposure may have on cognitive functions such as memory, particularly in children and adolescents. In response, the WHO recommended: (a) conducting prospective cohort studies of children and adolescents with outcomes including behavioural and neurological disorders, and (b) quantifying personal exposures from a range of RF sources and identifying the determinants of exposure in the general population. Accomplishing these objectives has been difficult due to the constantly evolving issues in RF-EMR exposure assessment (dosimetry), which have limited progress in epidemiological research.

Mobile phone calls are the largest source of RF-EMR exposure, especially to the brain [6]. Therefore, mobile phone use forms the primary exposure metric in most brain tumour and cognitive function studies. The pattern of use of mobile phones has changed greatly over the last 10 years with the introduction of smartphones. By June 2016, an estimated 76% of Australian adults used a smartphone compared to just 49% only five years earlier [7]. Use of fixed-line telephones services has dropped from an estimated 78% of Australian adults to 68% in the same time frame, while over-the-top (OTT) communication services such as Whatsapp, Messenger, Viber, Skype and Facetime have steadily increased in practice to an estimated 25% of Australian adults. The volume of data downloaded from mobile handsets has increased by 69% in the last year, which is consistent with trends observed elsewhere in countries such as the USA and the UK [7]. These rapid changes to the exposure of the general population over the last decade have cast doubt on the generalisability of the results from previous studies. 

Many of the previous studies were prospective cohort studies investigating the possible association between RF-EMR exposure and memory performance, but they have produced contradictory findings. The Mobile Radiofrequency Phone Exposed Users (MoRPhEUS) study was the first to investigate the effects of mobile phone use and memory, as well as other cognitive functions, on adolescents in Australia [8]. The cross-sectional analysis found that the accuracy of working memory was poorer in 7th grade students (median age 13 years) who reported making more voice calls with their mobile phone. However, this result was also observed in children who reported sending and receiving more SMS messages as well, suggesting that these findings were due to behaviour learnt through frequent mobile phone use and not from RF-EMR exposure. 

The follow up study one year later found that participants who had a higher number of calls and SMS texts at baseline showed less of an improvement in the two-back working memory task [9]. Yet participants who experienced an increase in voice calls and SMS texts from baseline to follow up showed a reduction in response times for the two-back working memory task. As these findings were seen in participants with more voice calls and SMS texts, it was considered unlikely to be due to RF-EMR exposure as very little RF-EMR was emitted during text messaging. However, the follow up time for this analysis was relatively short for dose-related changes in cognitive function in adolescents, and the exposure quantification for the study was dependent on self-reported mobile phone use, which has been shown to involve varying levels of uncertainty [10,11,12,13].

The more recent Health Effects Related to Mobile phonE use in adolescentS (HERMES) study conducted in Switzerland [14], used a comprehensive exposure surrogate consisting of mobile phone call duration, objectively recorded operator data, and propagation modelling [6]. With 7th-to-9th grade (mean baseline age 14) students followed for a single year. Strong associations were found between RF-EMR doses and figural memory, but not for verbal memory. The authors speculated this could be due to the different regions of the brain used for figural and verbal memory, as figural memory related activity is seen more predominately in the right hemisphere of the brain [15], and 81.2% reported using their mobile mainly on the right hand side. While verbal memory related activity is seen more predominately in the left hemisphere [16], and only 18.8% of study participants reported using their mobile on the left hand side.

The Examination of Psychological Outcomes in Students using Radiofrequency dEvices (ExPOSURE) study, which was conducted with 4th grade primary school students who had a mean age of 9.9 years also relied on self-reported questionnaires on mobile and cordless phone use for exposure assessment. These questionnaires were completed by the participants’ parents and follow up was conducted a year later. Weak evidence was found of an association with the higher usage of mobile phone group having lower accuracy for visual recognition memory in the cross sectional analysis [17]. However, the longitudinal analysis did not find any further evidence of an association between RF-EMR exposure and visual recognition memory. This led to the conclusion that this association was only a chance finding [18]. 

It is difficult to directly compare the MoRPhEUS, ExPOSURE and HERMES studies as they have different exposure metrics and the outcomes were tested by multiple cognitive test batteries: CogHealth and the Intelligenz-Struktur-Test 2000R (I-S-T 2000R). They also all have various sources of uncertainty whose nature and magnitude were not analysed in great detail. Potentially these sources of uncertainty may be the primary cause of the inconsistencies seen between these epidemiological cohort studies and a more thorough uncertainty analysis could potentially reconcile these differences. The aim of this review was to identify the sources of uncertainty in currently used exposure assessment techniques and study designs in the MoRPhEUS, ExPOSURE and HERMES studies which investigate RF-EMR exposure and memory performance in children and adolescents. This review was limited to epidemiological cohort studies investigating RF-EMR exposure and memory outcomes in children and adolescents.

## 2. Uncertainty Analysis

Uncertainty has a negative connotation, as it is associated with a lack of conviction in the results and a widening of the uncertainty band or confidence interval [19]. However, uncertainty analysis is critical to obtaining more value from epidemiological data to aid in risk assessment and quantifying confidence in the results [20]. In the field of RF-EMR and cognitive function performance, there are a large number of potential sources of uncertainty. These sources include:

- Statistical variability
- Measurement error
- Context
- Expert opinion (subjective judgements)
- Linguistic uncertainty
- Model input uncertainty
- Model uncertainty

**Figure 1:**
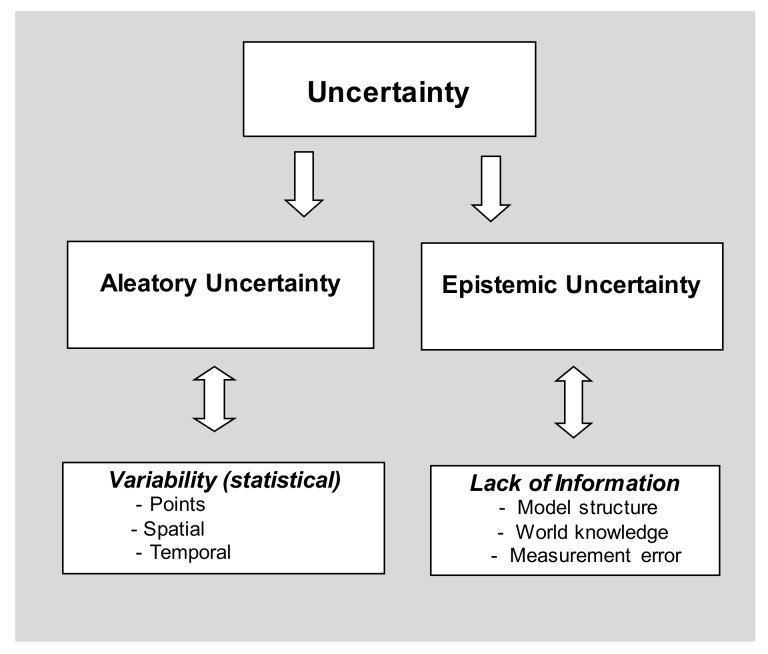
A taxonomy of uncertainty that shows the division between statistical variability and epistemic uncertainty.

These sources of uncertainty have been characterised into two distinct groups in previous studies [21,22]: aleatory uncertainty (statistical variability) and epistemic uncertainty (lack of information). Being the most common source of uncertainty, statistical variability is widely elaborated and accounted for (using confidence intervals and *p*-values) while epistemic uncertainties have not been widely discussed in the epidemiological literature. There has been a continuous development of the taxonomy of uncertainty analysis. A taxonomy of uncertainty is presented in Figure 1. 

Linguistic uncertainty is considered as a separate category by Regan et al. [23], as it covers ambiguity in speech and writing, interpretation and information loss in communication. In this paper it will be regarded as a source of epistemic uncertainty as categorised by others [24,25]. 

### 2.1. Aleatory Uncertainty

**Figure 2:**
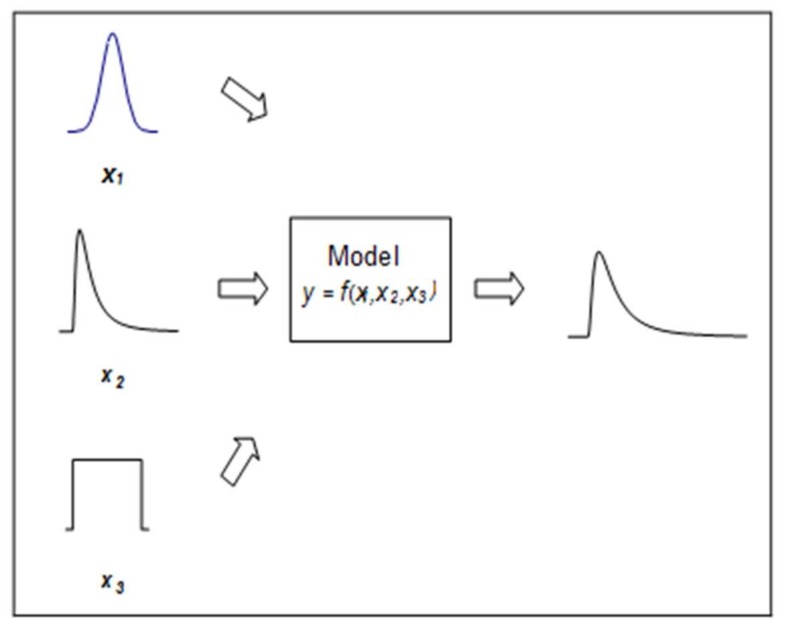
Monte Carlo simulation is an iterative process where each trial requires a new set of inputs which are random samples from probability distributions. After the simulation has been halted, the output of the model is represented by a probability distribution from which the mean, median and confidence intervals can be calculated.

Aleatory uncertainty is generally regarded as irreducible due to the fact that it is the natural statistical variability found in data. It is used interchangeably with the term “statistical variability” or “error”. It is regarded as irreducible because there will always be a residual error in a study. Aleatory uncertainty can be characterized via probability distributions, using replicated data, and quantified as the output of a model using a Monte Carlo simulation [25]. In Monte Carlo simulation, values are drawn at random from a probability distribution for each input; the model output is computed in an iterative process that produces an output distribution for the model. (See Figure 2).

Probabilistic simulation is a powerful method of addressing statistical variability and its propagation through a modelling process [26]. For example, uncertainty can be treated in the context of a dose response curve for input uncertainty, where the model may be inserted in the innermost loop of a Monte Carlo simulation [27]. 

### 2.2. Epistemic Uncertainty

Epistemic uncertainty is characterised as a source of uncertainty that is due to a lack of knowledge. It differs from aleatory uncertainty in that it is reducible with the collection of more information. For example, insufficient understanding of the underlying processes or imprecise assessments of exposure or outcome metrics are common sources of epistemic uncertainty, but these can be reduced by acquiring more knowledge. Epistemic uncertainty can be further classified into two types. Type 1 uncertainty is the most common type and relates to uncertainty caused by “known unknowns” such as data transcription errors and linguistic ambiguity. Measurement error, context, expert opinion and model uncertainty can also fall under this category. Type 2 epistemic uncertainty refers to “unknown unknowns” and is much less common, but far more serious and potentially disastrous. These events are often very rare, unexpected and can have potential major impacts [25]. Biological examples include the Spanish flu of 1918 or the Bubonic Plague of 1349. Salient differences between epistemic and aleatory uncertainty are summarised in Table 1.

Common sources of epistemic uncertainty that may occur in epidemiological studies investigating RF-EMR exposure and memory performance are detailed below:

### 2.3. Measurement Error

Measurement error is classified as epistemic uncertainty because it can be reduced by more precise measurement due to superior instrumentation, improved experimental techniques and minimisation of extraneous environmental factors. The skill and experience of a subject matter expert may also influence the result. However, there will always be a residual random error that is irreducible due to natural variability [25]. 

Adding further to epistemic uncertainty in measurement are the large number of common devices emitting RF-EMR that now exist in the environment. These include mobile and cordless phones, base stations, FM radio, smart TVs, Wi-Fi routers, laptops, tablet computers etc. Personal exposure from these devices can be categorised as either near-field or far-field. Near field sources, such as mobile and cordless phone calls, operate in close proximity to the head or body and result in sporadic RF-EMR exposure. Far field sources, such as base stations and Wi-Fi, occur from much greater distances away, resulting in a much lower but constant level of RF-EMR exposure. Near field sources account for the majority of RF-EMR exposure, with mobile phone calls responsible for the largest amount of RF-EMR to the brain [6], but two users who make the same number and duration of calls can have vastly different levels of exposure. The distance each person holds the phone from the head greatly changes the exposure level. For instance, a mobile phone call taken approximately 2.5 cm away from the brain is considered the standard exposure, but when the phone is moved to a distance of 5 cm away there is an 89% exposure reduction, at 10 cm the exposure reduction is 96% and at 25 cm 99% [28].

If a person uses an earphone or loud-speaker the RF exposure from a mobile phone call becomes virtually negligible. Signal strength and type of phone also greatly affect the amount of RF-EMR exposure, as a poor signal requires the phone to use more power and increases exposure, while different phone types have different Specific Absorption Rate (SAR) limits and different locations of antennae varying the level of exposure received. Laterality of phone use also needs to be considered, but self-reported laterality has been found to have limited validation [29]. The SAR is a measure of the rate of RF energy absorbed by the body in terms of Watts per kilogram averaged over a small sample of tissue, usually 1 or 10 g. This information is very difficult, if not impossible, to collect in the field with current exposure methods. 

Questionnaires derived from the Interphone study only asked the number of calls and texts sent and received, as well as the duration of the calls, but this has been shown to be inaccurate [10,12]. Participants have also been found to consistently underestimate the number of calls made and received per month, while overestimating the duration of each call [11]. This is a case of epistemic uncertainty, where more information could decrease this source of error.

Billing records provide more thorough and accurate mobile phone usage data, but lack key information such as distance from head, signal strength, laterality and phone calls made through OTT services. There is also the possibility that other users have accessed the phone which cannot be accounted for with billing records. Errors in documentation represent a source of epistemic uncertainty.

Far field sources account for some exposure to the brain, but contribute a much lower proportion compared to near field sources. Base stations are the largest source of far field RF-EMR exposure, while Wi-Fi, TV and radio make significantly smaller contributions. Distance from a base station at work and at home is a poor proxy as a clear line of sight, vegetation, reflective walls and building materials can result in a large amount of misclassification [30]. However, the 3D radio wave propagation model NISMap has been found to provide accurate measurements of electric field strength from mobile phone base stations in complex urban environments for fixed locations [31]. This requires antenna height, location, direction, frequency and a box model of 3D building data to be accurate for fixed locations [32], which may not be the best model for exposure assessment in users who spend a lot of time away from the office, school or home. 

An alternative is exposimeters or exposure monitors, which are able to estimate personal far-field exposures while being worn by a participant. This is advantageous because it provides a more accurate measure for a moving participant. However it is still prone to significant measurement error. Firstly, the sensitivity at the lower detection limit may not be high enough to detect low RF-EMF for some frequencies in many environmental settings [33]. Secondly, participants are required to carry the exposimeters around, so it is imperative that they are small and light. Otherwise, it may be difficult and inconvenient for participants to carry them around for long periods of time resulting in low participation rates. Finally, measurements recorded by exposimeters worn on the body have been found to be underestimates due to body shielding, which would randomly reduce the level of RF-EMR exposure recorded [34,35]. 

### 2.4. Context

**Figure 3:**
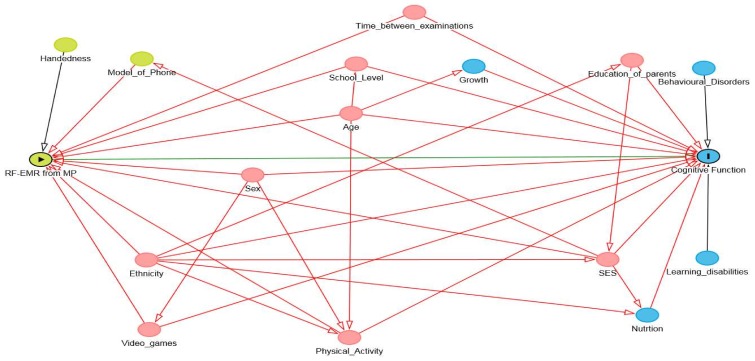
An example of a directed acyclic graph for associations between RF-EMR exposure and cognitive functions. Green variables are ancestors of the exposure. Blue variables are ancesters of the outcome and red variables are ancesters of both exposure and outcome.

Recognising the correct context, which is the external environment and conditions that affect the association being investigated, is important for reducing the uncertainty in the models. Failure to account for confounders and effect modifiers can introduce significant bias into the model leading to false findings. This is particularly difficult in RF-EMR exposure research, as the purpose for using RF-EMR emitting devices is changing frequently. Economic and social factors need to be considered and represented in the model. The Directed Acyclic Graph (DAG) is an effective method for identifying confounders and effect modifiers as well as the structure of associations between variables of interest [36]. DAGs are transparent illustrations of the causal hypothesis which can help the author rationalise analytical methods used in a study. They can also be used to identify methodological problems that could introduce uncertainty into the model. DAGs have been recommended for broader use, and to be included in supplementary material by journals [37,38]. As the context for RF-EMR is constantly shifting, it is important to clearly document the assumptions made and associations tested to prevent potential sources of uncertainty from entering the model. Figure 3 shows an example of a DAG developed from the adjusted variables found in the MoRPhEUS, ExPOSURE and HERMES studies. Based on this DAG, age, ethnicity, physical activity, socio-economic status (SES), school level, sex, time between examinations and video gaming would need to be adjusted for to estimate the direct effect between RF-EMR exposure and cognitive function outcomes. However as further information is gathered, this can be adjusted to reflect changes in the covariate structure.

### 2.5. Expert Opinion (Subjective Judgement)

A potential source of epistemic uncertainty can occur when subjective judgement is used to estimate facts or classifications. For example, subjective judgements are made in these studies regarding the age of participants and the length of follow up time. Investigators may have picked the age and follow up time where they believed they were most likely to see an association had one existed or due to logistic and funding constraints. However, as previously mentioned, a one year follow up is a relatively short period for dose-related changes in cognitive function in adolescents. Also the age at which adolescents start using a mobile phone and other RF-EMR emitting devices has changed over the past decade with exposure now occurring at a younger age compared to a decade ago. Data interpretation can also be considered a subjective judgement. Only with further investigation could uncertainty arising from these subjective judgements be reduced.

### 2.6. Linguistic Uncertainty

Linguistic uncertainty is possible because any language is vague and ambiguous. It is considered epistemic because it can be reduced by resolving ambiguities and increasing specificity. Ambiguities are common because some words have multiple meanings and interpretations. Australia has a multicultural society and different ethnic groups could have different interpretations to the same word. There is also a strong chance that English may not be the first language for a significant portion of participants in Australian studies. In some European countries there are many different dialects spoken within the same city and in the broader field of mobile phone use and health, the Interphone and Mobi-kids studies were conducted in multiple countries [4,39,40]. Even though the questionnaires used were validated and locally adapted, the possibility of some data being ‘lost in translation’ cannot be dismissed. This could theoretically introduce a systematic bias that would skew the findings from this study.

### 2.7. Model Input Uncertainty 

**Figure 4:**
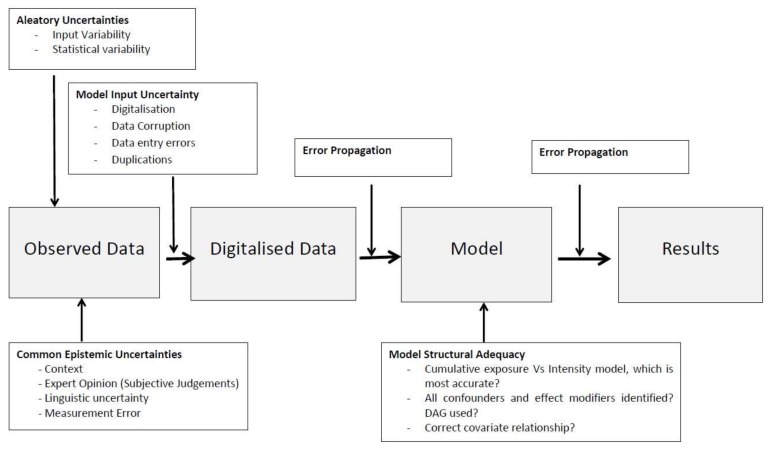
Visual representation of the sources of uncertainty found throughout the modelling process of RF-EMR and Cognitive function. Sources of uncertainty can enter the modelling process at different points and can be due to known and unknown sources. (Adapted from Robinson et al. [25]).

The digitisation of raw data and the transfer of data into spreadsheets, or other software packages, are inputs that are subject to errors. Data corruption, entry mistakes and duplications are examples of input errors which can propagate through the model and contribute to output error [26]. This is considered an epistemic source of uncertainty as it can be reduced with more thorough data checking and cleaning. Sources of error and the subsequent error propagation during the modelling process are shown in the schematic Figure 4.

### 2.8. Model Uncertainty

Where possible, dose-response associations should be investigated, however, because it is extremely difficult to measure the dose for RF-EMR, many exposure metrics are used as proxies for dose. Therefore, exposure-response associations are investigated instead. The exposure-dose relationship can introduce uncertainty into the model, as it is difficult to know what summary measures of exposure accurately reflect the biologically effective dose a priori [41,42]. The most common exposure summary measures are duration of exposure, exposure intensity and cumulative exposure [43]. Cumulative exposure is the most frequently used metric in the field of RF-EMR research and is commonly used in other fields for chronic diseases with long latency [43], as it has been shown to correlate more strongly in cancer risk studies [44].

However, cumulative exposure models may not be the most appropriate for non-cancer related outcomes, such as cognitive function and memory performance, or for doses which have a rapid biological clearance rate. So far, only tissue heating has been identified as a confirmed health effect from RF-EMR exposure. The levels experienced from mobile phone and other RF-EMR emitting devices are low and have been shown to result in a negligible temperature increase, as the human body is adept in thermoregulation [1]. Other pathological mechanisms cannot be ruled out, but research into RF-EMR exposure and the effect on brain electrical activity, sleep, heart rate and blood pressure have not found consistent evidence of adverse health effects at levels below those required to cause significant tissue heating [1]. As tissue heating can be quickly dealt with by thermoregulation, an intensity based model may be more appropriate. Cumulative exposure models assume that each unit of dose causes a constant amount of injury [45]. They also assume that continuous exposure and short duration high intensity exposure which average out to the same amount of cumulative exposure are independently related to disease risk [41]. These assumptions introduce uncertainty into the model which can only be reduced with further research.

## 3. Uncertainty in RF-EMR and Memory Research in Children and Adolescents

Critical appraisal of the MoRPhEUS, ExPOSURE and HERMES cohort studies [9,14,18] investigating RF-EMR and memory outcomes shows significant sources of uncertainty that were not addressed, or were qualitatively, but not quantitatively discussed. This is common across all epidemiological cohort studies as well as cross sectional studies investigating other cognitive outcomes such as the Amsterdam Born Children and their Development (ABCD) study [46], as the potential for bias is usually discussed qualitatively but rarely quantified [47]. 

### 3.1. The MoRPhEUS Study

The MoRPhEUS study was the first of the cohorts conducted which found working memory to be poorer in the cross-sectional analysis, but no associations were found in the longitudinal analysis. This study only addressed attrition bias quantitatively. This was done by considering the participation rate (75%) and comparing the baseline demographic, exposure and cognitive function data between those lost to follow up and those who participated to show no significant differences. Measurement error was qualitatively discussed by acknowledging self-reports were prone to awareness bias and misclassification [48], but was justified as the questionnaire was based on a validated instrument derived from the Interphone study [49]. The exposure metric used in this study was number of calls made and received, not cumulative duration as self-report of this had been shown to be more inaccurate in adolescents [10]. This may have reduced measurement error in the recorded exposure data, however the proxy used for exposure is far from representative of the biologically effective dose to which each participant is exposed. Therefore, non-misclassification was still a major source of uncertainty in the study. 

To adequately quantify RF-EMR exposure from a mobile phone would require data on duration, signal strength, laterality, phone type, distance and use of loud-speaker to be recorded, but practical and reliable means of recording such were not available at the time for signal strength, laterality and distance. Objectively recorded mobile phone billing records were unable to be collected which would have provided a quantitative assessment on the self-reported data for both number of calls made and received as well as duration of calls. Consequently, the exposure proxy introduces a significant source of uncertainty into the study. There are also additional sources that need to be addressed. 

Uncertainty introduced via linguistic ambiguity was not discussed in the MoRPhEUS study. However, 26% of the participants at the study baseline spoke a language other than English at home and 16% were born in a country other than Australia. The mean age at follow-up for the participants was 13.8 years. It is therefore conceivable that some did not interpret the questionnaire in the manner in which it was intended. However, to be included in the study, the student and their parent or guardian had to be able to understand the information on the plain English language information packages and consent form; subsequently the linguistic ambiguity uncertainty would be expected to be minimal. 

A DAG was not presented in the publications of the MoRPhEUS study, which introduces uncertainty to the context between the exposure and outcome association, and the structure of associations between variables of interest. Notably, computerized or video gaming was not adjusted for. Although only speculative, experience of computerized gaming could increase the learning effect seen in the computerized cognitive test battery resulting in apparently better cognitive function scores. Previous studies have found older participants who partake in frequent console gaming to have improved cognitive performance for: change detection [50], visual attention and inhibition [51,52], working memory and spatial memory [53,54]. There is uncertainty regarding the subjective judgement for length of follow up time as it may not be long enough to see an association. 

Furthermore, there was uncertainty of the model structure adequacy. Two models were used: the first was changes in cognitive function outcome versus exposure at baseline which was done to test if there was a latent effect. The second was whether an increase in exposure resulted in a change in the cognitive function outcomes [9]. Neither of these models tested cumulative exposure or exposure intensity which would be more accurate representations of the exposure—dose relationship. Statistical variability and model input uncertainty could also be identified as sources of uncertainty in the MoRPhEUS study, but the predominant influences were measurement error and model structure uncertainty which would cause a significant widening of the confidence limits as these sources would bias the results towards the null. 

### 3.2. The HERMES Study

The HERMES study, which used the most complex exposure surrogate, also qualitatively discussed numerous sources of uncertainty [14]. The authors acknowledged the large amount of uncertainty in the dose measure calculations, recognizing that it was too difficult to quantify that source at the time of publishing and detailed some of the difficulties previously discussed under measurement error. Objectively recorded data were acquired for a subgroup of the cohort in the form of billing records, but even these were not without uncertainty. As the authors acknowledged, it was possible for someone other than the participant to make a phone call using the participant’s mobile phone and there was no validated method for converting data traffic volume into an effective biological dose measurement. Model uncertainty was briefly mentioned with a cumulative exposure model used, while the possibility of missed confounders was also discussed despite numerous potential confounders being considered. 

A DAG was not presented in the HERMES study publications, so the reader could not interpret the context between the exposure and outcome association, or the structure of associations between variables of interest. Selection bias was quantified as a participation rate, although loss to follow up was minimal with only 3.2% of the population failing to return after baseline measurements. Yet initial recruitment for the cohort was described as ‘moderate’ which as discussed in the publication may impact the representativeness of the findings to the broader population. Only relatively minor sources of uncertainty such as model input and statistical variability were not mentioned. Importantly the authors acknowledged that the positive results need to be interpreted with caution given the complex correlation structure for various exposure measures and the uncertainty in the RF-EMF dose calculations. The HERMES study provides the most thorough uncertainty analysis of all the epidemiological cohort studies conducted on RF-EMR and cognitive function. Yet due to limitations in the field of exposure assessment, it was still unable to quantify measurement error which is the largest source of uncertainty in the study, despite acquiring some objectively recorded billing records.

### 3.3. The ExPOSURE Study

The recent ExPOSURE study had similar sources of uncertainty to the MoRPhEUS study, but was conducted with a younger and larger cohort of children. As in the MoRPhEUS study, selection bias was addressed quantitatively by the participation rate (66.5%) and comparing the baseline demographic, exposure and cognitive function data to show no significant differences other than a higher SES in those who participated compared to those who dropped out. Measurement error was also qualitatively discussed as a modified validated questionnaire was used to collect data on the number of mobile and cordless calls made and received [49]. This has the same potential sources of uncertainty previously mentioned above, but also an additional factor which is the parents/guardians of the participants were asked instead of the participants themselves [55,56]. A relatively simple question asked to both parents/guardians and the participants found quite different results. For the question: “Does your child use or own a mobile phone?” 31% of parents/guardians responded affirmatively at baseline compared to the participants who self-reported a figure of 57%. This difference was also seen when reassessed at follow up with 43% of parents and guardians reporting ownership or usage compared to 68% by the participants themselves. If this difference was consistent across the exposure assessment questions, then the potential for differential misclassification was a large source of uncertainty. 

The other major source of uncertainty was in the model. One of the models used distinguished exposure based on whether there was an increase or no increase in the number of calls made and received between baseline and exposure. This created a scenario where, for example, it was possible for participant A to average 4 calls at baseline and 4 calls at follow up while participant B had 0 calls at baseline and 2 calls at follow up yet participant B ended up in the exposure of interest category. The second model was more of a cumulative exposure model with the participants split into 3 categories: ‘None’ (Never used or owned a mobile phone), ‘Some’ (below the median of use in those who use a mobile phone) and ‘More’ (above the median of use in those who use a mobile phone). This model introduced expert opinion uncertainty as the median split classifications may not be appropriate to identify an association. In comparison; the Interphone study split the participants into deciles and an association was only seen in the highest decile for cumulative exposure [4]. However, the ExPOSURE study was unable to categorize the participants into deciles as they had significantly fewer participants compared to the Interphone study. 

Another source of uncertainty found is linguistic. This is seen in the questionnaires given to participants in the study. In the questionnaire used to quantify RF-EMR exposure from mobile phones, lack of specificity can be found. The participants were asked how many mobile phone calls they made and received in a week, but the questionnaires did not specify whether calls made through OTT services like WhatsApp, Viber or Skype should be included. This was also the case with SMS texts; there was no specification on whether messages sent through OTT services should be included or excluded. These services were only introduced a year or two (Whatsapp and Skype in 2009, Viber in 2010) before the ExPOSURE study was conducted (mid-2011) and were not then as popular. These services were also only available on smartphones so therefore likely linguistic uncertainty introduced via lack of specificity was minimal. Other minor sources of uncertainty not discussed in the ExPOSURE study were model input uncertainty and aleatory uncertainty. There was uncertainty on the correct covariate relationship as a DAG was not presented. 

### 3.4. The ABCD Study

In addition to these cohort studies, there is also a cross sectional study from the Amsterdam Born Children and their Development (ABCD) study in The Netherlands [46]. Although it did not test associations between sources of RF and memory, it did look at other cognitive functions via the validated Amsterdam Neuropsychological Tasks program [57], such as speed of information processing, inhibitory control, cognitive flexibility and visuomotor coordination. Results were inconsistent, with children exposed to higher levels of RF from base stations and residential sources having improved inhibitory control and cognitive flexibility, while children with a higher cordless phone use had a reduced inhibitory control and cognitive flexibility. This study has similar sources of uncertainty that the previously described studies experienced. Measurement error was a significant problem. The 3D geospatial radio wave propagation model NISMap was an effective method used to calculate exposure from base stations, but these contributed only a small proportion of the total RF-EMR dose. The more significant cordless phone use was measured via questionnaire. Other sources of uncertainty such as model uncertainty were also significant concerns and could explain the inconsistent findings observed. A summary of these studies is shown in Table 2.

## 4. Discussion

This review has investigated the concept of uncertainty analysis in relation to the field of RF-EMR and cognitive functions, particularly with respect to memory performance in children and adolescents. It was necessary to summarise, characterise and interpret many sources of uncertainty present, especially in exposure assessments which are extremely difficult to quantify objectively. In analysing the previous studies, it has been found that predominantly only a qualitative assessment of sources of uncertainty was conducted, yet it is imperative to conduct more robust quantitative uncertainty analysis to strengthen the findings of these epidemiological cohort studies and improve the usefulness of epidemiological data for risk assessment. 

The primary sources of uncertainty across these studies on RF-EMR exposure and memory are measurement error and model uncertainty. It is possible that by reducing these sources of uncertainty the conflicting results in memory performance could be reconciled. New objective methods need to be developed to refine exposure assessment, as billing records have become difficult to acquire in some countries such as Australia and do not provide all the necessary information. For instance, translating data traffic volume to a biologically effective RF-EMR dose has become a necessity as 25% of Australian adults now use OTT services and the proportion is rising [7]. 

Mobile phone applications (apps) such as XmobiSense and QuantaMonitor provide a potential alternative for exposure assessment [58]. The XmobiSense app has been used in the Mobi-Kids study and has been useful in pilot studies [12,39,40]. The XmobiSense app provides data on: The number of calls and SMS, duration of use, laterality, receiving power (Rx) of the mobile network and Wi-Fi (dBm), amount of transmitted and received data (kbs). However, it does not collect data for Transmission power (Tx). The QuantaMonitor App measures both Tx and Rx power from mobile phones and Wi-Fi networks and uses an algorithm to calculate the SAR. These apps provide data that cannot be obtained through self-report or billing records, however further investigation is required to assess the accuracy and reliability of data provided from both Apps. It is important that the validity of the output of the Apps be determined, since it would be a waste of resources to use unreliable or inaccurate Apps in future population studies. This could potentially be done by the use of Engineered Phones, such as the Qualipoc Handheld Android (SwissQual, Munich, Germany), which is programmed to provide accurate data from the electronics of a mobile phone.

The model uncertainty observed in these studies could in part be due to uncertainty over the biological mechanisms through which RF-EMR causes disease. The only established negative health effect from RF-EMR is thermal heating, however despite little and inconsistent evidence, non-thermal effects cannot be dismissed entirely. Yet without establishing a biological mechanism for disease via non-thermal effects, the model will always be under some degree of epistemic uncertainty. 

In the context of RF-EMR exposure and cognitive function, it is biologically plausible that cognitive functions associated with the temporal lobe of the brain such as memory, visual item recognition and auditory processing are more likely to have an association compared to functions associated with regions deeper in the brain that receive less exposure from handsets. The amount of exposure decreases with distance, which needs to be taken into account, as well as the debate over cumulative exposure vs. intensity model. This source of epistemic uncertainty could only be reduced with further research, but the broader usage of DAGs and Monte Carlo simulation could certainly be used to improve uncertainty analysis by identifying the correct covariate relationships and providing probability distributions to represent variables with inherent uncertainty. 

There is also the possibility that the associations observed within these studies between RF-EMR and memory could be due to behaviours learnt from mobile phone use and not from RF-EMR exposure per se. Some studies have found that smart phone related behaviours could be detrimental to memory function [59]. Sparrow et al. found that participants were remembering less information and instead memorizing where that information could be found concluding that “the processes of human memory are adapting to the advent of new computing and communication technology” [60]. Uncapher et al. showed a reduced working memory ability for those who used a greater frequency of media multitasking [61]. 

Another important consideration required when interpreting these conflicting results is the cognitive test batteries used. The MoRPhEUS and ExPOSURE studies use the validated computerized test battery CogHealth [62,63,64,65], while the HERMES study used the validated computerized test battery Intelligenz-Struktur-Test (IST) [66]. The CogHealth battery used a one and two card back task to assess working memory which was different to the IST tasks for figural and verbal memory. Verbal memory was tested by word groups being memorized in a minute before giving an account of the word groups memorized. The figural memory task use involved pairwise symbols being memorized after a minute one of the symbols is shown and the other matching part has to be found. Although these tasks have been validated for use on children and adolescents [62,63,64,65,66], and the measurement error minimal, the differences between the tasks used mean caution is required when comparing the results between these studies.

## 5. Conclusions

Epistemic uncertainties include errors in human judgement, transcription, measurement, model structure and data processing errors. These uncertainties are often not appreciated by researchers and make it difficult to produce definitive conclusions from a single study of the effect of RF-EMR exposure on memory. It is important to consider uncertainty analysis in order to strengthen confidence in findings and to increase the accuracy of risk assessment. 

Specifically, in addition to replicating statistical studies, a more comprehensive analysis of possible error sources could be made by supporting consideration of epistemic uncertainty. Once the major epistemic errors have been identified, the next step is to quantify these errors where possible, which would aid in their reduction and enable treatment in combination with errors due to statistical variability. A well-established method in the peer-reviewed literature is Monte Carlo simulation, which has the advantage that it can combine different sources of uncertainty into a total figure for uncertainty by representing model inputs with probability distributions for both statistical variability and quantified epistemic uncertainty [24,25].

## Figures and Tables

**Table 1:** Key differences between aleatory uncertainty and epistemic uncertainty (adapted from Benke et al. [21]).

Aleatory Uncertainty	Epistemic Uncertainty
Stochastic	Subjective
Irreducible	Reducible
Variability	State of Knowledge

**Table 2:** Summary of studies investigating RF-EMR and cognitive function.

Study	Design	Number of Participants (N)	Age (at Baseline)	Cognitive Test Battery	Memory Outcome	Associations Found
MoRPhEUS	Cohort	Baseline: 317 Follow-up: 238	12.9 (11.7–14.3) ^1^	CogHealth	Working Memory	One back task ^4^ −0.091 (−0.170, −0.013) Two back task ^4^ −0.098 (−0.169, −0.027)
HERMES	Cohort	Baseline: 439 Follow-up: 425	14 (0.85) ^2^	Intelligenz-Struktur-Test 2000R	Figural and Verbal Memory	Figural memory ^5^ Brain: −1.16 (−1.99, −0.34) Body: −0.86 (−1.67, −0.05)
ExPOSURE	Cohort	Baseline: 619 Follow-up: 412	10 (0.4) ^2^	CogHealth	Working Memory	None
ABCD	Cross sectional	Baseline: 2354	(5–6) ^3^	Amsterdam Neuro-psychological Tasks program	None	N/A

^1^ Interquartile range; ^2^ Standard deviation; ^3^ Range; ^4^ Arcsine transformed accuracy. Regression coefficient (95% confidence interval) between total reported voice calls per week and working memory from cross sectional analysis; ^5^ Figural memory performance in highest exposure category (≥75%) compared to lowest (≤50%) by dose measurements. Regression coefficient (95% confidence interval) from longitudinal analysis.
